# Do Nonhumans Seek Explanations?

**DOI:** 10.26451/abc.07.03.10.2020

**Published:** 2020-08-01

**Authors:** Christoph J. Völter, Megan L. Lambert, Ludwig Huber

**Affiliations:** 1 Messerli Research Institute, University of Veterinary Medicine Vienna, Medical University of Vienna, University of Vienna, Vienna, Austria; 2 University of St Andrews, St Andrews, UK

**Keywords:** Explanation seeking, Hypothesis testing, Chimpanzees, Causal cognition, Folk physics, Object exploration

## Abstract

From an early age, children explore their environment in a way suggesting that they reason about causal variables and seek causal explanations. Indeed, following extensive studies of problem-solving abilities in chimpanzees, Povinelli (*Folk Physics for Apes*, Oxford University Press, 2000) proposed that this ability to reason about unobservable variables is unique to humans. Following on from this, Povinelli and Dunphy-Lelii *(Canadian Journal of Experimental Psychology*, 55(2), 187–195, 2001) addressed the question whether chimpanzees would explore objects with the aim of elucidating unobservable and surprising object properties. Chimpanzees, unlike preschool children, did not show increased object exploration following a change in the unobservable properties of an object. We critically discuss these findings and argue that more research using a greater variety of methods and with a larger number of species is required to support the hypothesis that only humans engage in explanation seeking. We conclude by highlighting avenues for future research based on developmental and comparative research aimed at object exploration and information seeking conducted since the original investigation by Povinelli and Dunphy-Lelii.

Most contemporary comparative cognitive research has been devoted to the question what kind of information animals represent and to what extent they can make inferences about their (physical and social) environment based on these representations (for recent reviews, see Seed & Mayer, 2017; Völter & Call, 2017). In the literature on chimpanzees’ physical cognition, Daniel Povinelli’s *Folk Physics for Apes* (2000) has been an influential representative of this line of research focusing on the question whether chimpanzees (*Pan troglodytes*) reason about unobservable variables such as weight or others’ mental states to generate predictions about the events they observe in their environment.

Inferential reasoning has been classified into predictive inferences (reasoning from a cause to its effect) and diagnostic inferences (reasoning from the effect to its cause, sometimes also referred to as postdiction; Visalberghi & Tomasello, 1998). Whether nonhuman animals engage in diagnostic causal reasoning has been particularly controversial (e.g., Penn & Povinelli, 2007; Völter & Call, 2017). This type of diagnostic reasoning would allow generating explanations about causal relations in the environment. Whereas the adaptive value of forward predictive reasoning is typically taken for granted, it is less apparent why animals should engage in diagnostic reasoning from an evolutionary point of view.

Closely related to diagnostic causal reasoning is the question whether animals actively seek explanations, that is, to what extent they search for information to elucidate causal relationships. This may seem an odd question for those who associate the term “explanation” with the everyday (linguistic) meaning of (verbal) statements made to clarify the causes of some facts. Indeed, it has been suggested that diagnostic reasoning about unobservable variables and explanation seeking might be linked to natural language or symbolic representations (e.g., Penn et al., 2008; Vonk & Povinelli, 2012). However, although it seems natural to think we need language to generate explanations, at least the underlying motivation may simply be the resolution of a wondering why state as a response to an affective tension, such as a state of curiosity. Andrews (2012) has described the paradigmatic folk psychological explanation in terms of drives to relax the affective tension or to reduce cognitive dissonance.

Allowing a motivational impetus for explanation seeking opens the door to study it in nonhuman animals. Even if nonverbal organisms are not necessarily out of the game with respect to explanation seeking, whether they actually engage in this type of information seeking remains an open question. And, at the empirical level, we have to solve the problem of how to study it in nonhuman animals without asking them. First of all, this requires a precise definition that helps to prevent terminological confusion. Specifically, we need to find behavioral symptoms to distinguish between information seeking^1^ driven by a desire to uncover what is new or uncertain (novelty seeking, information sampling), to gain general information (information search; Gottlieb & Oudeyer, 2018), or to resolve knowledge-inconsistent phenomena or events (explanation seeking; Povinelli & Dunphy-Lelii, 2001; Stahl & Feigenson, 2015).

For comparative and developmental research involving nonverbal (or preverbal) participants, we can operationalize “explanation seeking” as a form of active information seeking that (1) is triggered by knowledge-inconsistent events (as opposed to novel, uncertain or unknown events), (2) is not directly reinforced, and (3) can involve interventions suitable to produce additional information concerning the knowledge-inconsistent event (see Stahl & Feigenson, 2015). Hypothesis testing, which requires producing interventions suitable to disambiguate causal relations in the environment, would be the prime behavioral indicator of explanation seeking. This is not to imply that explanation seeking can be triggered only by one or all of these criteria in combination; but rather, to provide guidelines for experimental paradigms that might allow interpreting information seeking behavior as explanation seeking.

Andrews (2005) argued that such “explanatory” paradigms (requiring to seek additional information when presented with the anomalous behavior of an agent or object) might be better suited to examine reasoning about unobservable variables in nonhuman animals than predictive paradigms (requiring to anticipate the effects of own actions or the behavior of an agent). This argument has been made in the context of mental state attribution but it can be extended to causal reasoning. The interpretation of results from predictive paradigms has often been controversial (associative learning vs causal reasoning, behavioristic vs mentalistic interpretations). Results based on the explanatory paradigm might be more difficult to explain based on associative learning and behavioral generalizations, precisely because of the focus on anomalous events to elicit a response (Andrews, 2005).

We can contrast our operational definition of explanation seeking with definitions of curiosity in the animal cognition literature. Accordingly, curiosity entails that animals are willing to give up a reward to acquire information, that no immediate benefit can be gained through the information, and that the tendency to seek information depends on the amount of information that can be obtained (Wang & Hayden, 2019). As mentioned before, curiosity might be an important motivational prerequisite for explanation seeking but not all curiosity results in explanation seeking (e.g., novelty seeking).

Explanation seeking paradigms typically involve events that are inconsistent with expectations about the environment as triggers for the information-seeking tendency. These expectations are based on prior knowledge, which is either drawn from core knowledge of object behavior—knowledge that is available from early in life or even innate (Spelke & Kinzler, 2007)—or from previous experience about object effects or object contingencies. If agents are sensitive to this conflict between what was predicted and what is observed, it becomes a scaffold for exploration. Recent research on human cognitive development shows that such violations are not just initiating information search, which refers to situations in which agents explore without prior knowledge of the task or goal (Gottlieb & Oudeyer, 2018). Rather, they are shaping preschool children’s exploration in a *targeted* way, which means they are learning only about objects relevant to the observed violation, seeking evidence that could explain the discrepancy between what was predicted and what is observed by identifying causal variables (Cook et al., 2011; Schulz & Bonawitz, 2007). In this sense, it may be justified to describe human children as scientists in the crib (Gopnik et al., 1999).

Interestingly, not only preschool children engage in this kind of ‘hypothesis testing,’ performing targeted actions to support or rule out possible explanations for a surprising event (Bonawitz et al., 2012; Legare, 2012), but also preverbal infants before their first birthday (Stahl & Feigenson, 2015). After witnessing surprising events that violated physical principles such as solidity and support, infants more readily learned a hidden auditory property of the object and preferred to manipulate these objects over new objects in a subsequent exploration phase. More importantly, the way they manipulated the test objects suggested they were testing hypotheses regarding the objects’ surprising behavior. For example, after witnessing a toy car moving over the edge of a surface without falling down, infants were more likely to drop this object to the floor than when they had witnessed a similar event in which the car did not move over the edge of the surface. When infants watched an event in which the toy car appeared to move through a solid barrier, infants were more likely to bang the car than to drop it compared to an event in which the car stopped in front of the barrier. In other words, infants’ exploration was suitable to produce additional information concerning the surprising object behavior. These findings can be interpreted as evidence that hypothesis testing does not presuppose verbal competence.

The question whether nonhuman animals also seek explanations followed from the research on causal reasoning presented in *Folk Physics for Apes* (Povinelli, 2000) and was explicitly investigated in a subsequent study by Povinelli and Dunphy-Lelii (2001). In this study, chimpanzees were trained over a period of six months to stand a block upright on a platform. The same task was also administered with 5-year-old human children that were shown by the experimenter how to set up the blocks together with a verbal instruction. Once the chimpanzees and children mastered this task, the experimenter covertly exchanged the blocks for visually identical sham blocks that could not be made to stand upright. The chimpanzees did not examine the sham block. Sixty-one percent of children, in contrast, inspected the bottom of the sham blocks at least once. Additionally, two of the 18 5-year-olds asked “Why?” and another two participants alluded to a physical cause for their failure. In a subsequent study using the same paradigm, children with autism spectrum disorder (ASD) were compared to typically developing children (Rutherford & Subiaul, 2016). The children in the ASD group showed higher exploration levels in some of the exploration categories (e.g., touching the surface of the table) and they offered significantly more physical explanations and asked more why questions.

Povinelli and Dunphy-Lelii (2001) concluded based on their findings: “it may be that our species alone develops an intrinsic interest in why objects have the properties that are apparent to the primary senses” (p. 194). In a more recent review article, Penn and Povinelli (2007) stated that “there is still no convincing evidence that nonhuman animals of any taxa seek out diagnostic explanations of anomalous causal relations or deliberately use their own interventions in order to elucidate ambiguous causal dependencies” (p. 110). In the following, we will argue that the comparative findings by Povinelli and Dunphy-Lelii need to interpreted with caution and that more research is required to support or disprove their hypothesis that only humans might engage in explanation seeking.

First, the task of placing a block in an upright manner (Povinelli & Dunphy-Lelii, 2001) might not be very intuitive and ecologically valid for chimpanzees. This notion is supported by the long training period that the chimpanzees required to pass the training criterion (about 20 sessions, in some cases longer). Relatedly, the way chimpanzees and children learned about the task goal differed fundamentally. Whereas chimpanzees learned the task goal over months through a shaping process; children received a short demonstration, verbal instruction, and two practice trials. The explicit setting of the task goal might have contributed to children’s exploration when this goal could not be reached in the test phase: for children the task goal of setting the blocks upright was presumably an active component of their working memory when they entered the test phase. The chimpanzees, in contrast, might have formed some procedural knowledge concerning the actions necessary to obtain the food reward during the training. That is, the chimpanzees might have encoded the necessary actions to obtain a reward over the course of their training, whereas the children might have primarily encoded the target state of the object (based on the verbal instruction). As a consequence, the chimpanzees might have attributed their failure in the test phase to their own actions and the children to the target object. It is an open question whether children would also have engaged in explanation seeking if they had learned about the task in the way chimpanzees had learned to perform the target behavior.

Second, the chimpanzees (unlike children) already had experience with other non-functional sham blocks (that were not visually identical to the training blocks; Experiment 1) before they were presented with the crucial experiment (Experiment 2) in which the functional and the sham objects were visually identical. This prior experience might have habituated them to the change in the object behavior, potentially reducing their motivation to explore the sham blocks in the critical test in Experiment 2 (Povinelli & Dunphy-Lelii, 2001). In Experiment 1, in which the training and sham blocks were not visually identical (the sham blocks had slightly beveled ends) the chimpanzees indeed showed levels of exploration comparable to 5-year-old children - a finding that Povinelli and Dunphy-Lelii discounted as a response to the subtle novel features of the sham blocks. However, it remains ambiguous whether perceptual novelty or an order effect explains the discrepant results between Experiment 1 and 2.

Third, curiosity and explanation seeking may be affected by early life experiences. For humans, there is evidence for cultural diversity in children’s explanation seeking (e.g., concerning the frequency of why questions; Gauvain et al., 2013). Moreover, the extent to which caregivers encourage explanation-seeking behavior might affect children’s tendency to show such behavior (Legare et al., 2017). Examining differences in information and explanation seeking across sites (including animals with different rearing histories and access to enrichment) might allow assessment of within-species variability. In particular, it might be interesting to study explanation seeking in highly enculturated animals. Rearing history and enculturation have also been highlighted as factors affecting the performance in physical cognition tasks such as the ones summarized by Povinelli (2000) in *Folk Physics for Apes* (Furlong et al., 2008; for a reply to this criticism see Povinelli, 2012).

Fourth, wider species comparisons are needed to warrant claims concerning human uniqueness. Obvious candidates here are other nonhuman ape species and, in general, tool-using species. Taken together, these points lead us to the conclusion that more research – with chimpanzees and also other species - is required to substantiate the conclusions drawn by Povinelli and Dunphy-Lelii (2001).

## Are There Other Paradigms That Have Been Used to Study Explanation Seeking in the Comparative Literature?

A recent study with kea parrots (known for their curiosity; *Nestor notabilis*) and New Caledonian crows (known for their tool using and manufacturing competence; *Corvus moneduloides*) addressed this topic in a different way. Specifically, the study examined whether the birds would adjust their object exploration to gain information about task-relevant object properties: Lambert and colleagues (2017) allowed the birds to explore a set of objects before and after they were presented with a foraging task in which some of these objects could be used as tools to extract a food reward. Which objects were functional, however, could be determined only by interacting with them (the functionality was determined by the weight or rigidity of the objects). A control group did not receive any opportunity to explore the objects. Individuals of both species chose the functional tool above chance but only if they had explored the objects before. Crucially, however, the duration and the way they explored the objects (i.e., the proportion of behaviors that could provide information regarding the relevant object property, such as lifting the objects) was not affected by their knowledge about the objects’ task relevance. That is to say, there was no significant difference between the first and second exploration phase (which took place before and after they were introduced to the task). Thus, even though the kea and New Caledonian crows acquired information concerning object properties during exploration, the study did not provide evidence that either species adjusted its exploratory behavior to the task requirements.

Outside of a problem-solving context, there is some evidence that at least one other species targets its exploration toward exploring invisible changes in objects, or objects that seem to violate some physical principle (e.g., where the center of gravity lies based on an object’s symmetry). Demery (2013) presented kakarikis (*Cyanoramphus novaezelandiae*) with a novel red ball and a familiar red rope to explore over three days. On the fourth day, the ball was replaced with a new object that differed in either its color, shape or its internal properties (i.e., a ball bearing was inserted inside so that the ball moved differently). Here subjects showed the greatest increase in exploration to objects that moved in a different manner rather than visually novel objects. In a second experiment, the same birds spent longer exploring novel objects that featured an unexpected center of gravity according to their symmetry; for example when symmetrical objects had an asymmterical center of gravity, or vice versa. These findings are consistent with the notion that karakiris form expectations about unobservable object properties such as the balance point of an object and explore objects more when these expectations are violated. However, given that these objects had no functional relevance (e.g., as tools) in this study, it remains a possibility that the karakiris are expressing a preference for certain haptic or kinematic object properties rather than an interest in why the objects changed their behavior.

## Future Directions

In summary, more research is needed to further examine the intriguing possibility that only humans seek explanations and actively test hypotheses concerning the behavior of objects and other agents. We suggested criteria that might help establish evidence for explanation seeking in nonhuman animals: information-seeking behavior triggered by knowledge-inconsistent events, not relying on direct reinforcement, and involving interventions suitable to provide more information concerning the anomalous event.

Future research might build on work from the developmental literature, using the violation-of-expectation paradigm (e.g., Stahl & Feigenson, 2015). In contrast to the paradigm administered by Povinelli and Dunphy-Lelii (2001), this paradigm does not involve a lengthy training protocol but it depends on expectations about certain physical principles (e.g., weight, solidity, and support). Relying on such pre-existing expectations might allow administering identical experimental protocols with human and nonhuman participants. Tasks involving long training protocols (and no verbal instructions), in contrast, are challenging to implement in developmental research. The first question will therefore be whether animals pay more attention toward objects or events that violate such physical principles. Research with dogs, primates, and corvids show that this paradigm is suitable in principle for comparative research (e.g., Bird & Emery, 2010; Davidson et al., 2017; Hauser & Spaulding, 2006; Müller et al., 2011; Pattison et al., 2010, 2013). If participants show an orienting response to the abnormal event, a subsequent exploration phase might indicate first whether animals show increased interest in this object and whether the way they explore the object is suitable to produce evidence explaining the unusual object behavior. Control conditions need to ensure that increased exploration rates are not triggered merely by perceptual novelty.

Other versions of this ‘broken mechanism’ paradigm could also be applied ideally using a more intuitive task. Such a task might require a direct causal link between the actions of the animal and the release of a food reward (e.g., a food dispenser that can be activated by inserting an object). If the mechanism fails in the test phase, it is important to show that the subject is not merely looking at the food reward but seeking information about the broken mechanism. A spatial separation between mechanism and the initial food location can resolve this issue. Alternatively, the animals might be presented with a change in the food-releasing mechanism. Even though the animals performed the same actions, the food is suddenly released in a different way. Would the animals seek additional information about what caused this change in the mechanisms despite having gained access to the food reward?

Finally, the paradigm established by Lambert et al. (2017) might be a useful starting point in the future to examine how object exploration in different species depends on the current task goals and the task-relevance of these objects. If object exploration is not affected by the task relevance of an object, this might provide further evidence for the absence of explanation seeking and hypothesis testing in a species (at least within a given test situation). Using a greater variety of methods with a more diverse group of participants (e.g., sanctuary-based or language-trained individuals) and species (in particular species that are known for their object play and tool use) will help us to understand whether we are indeed the only species that seeks explanations about the world.

## Footnotes

1 Information seeking has received increasing research attention in the last 20 years (for a recent review, see Marsh, 2019). These studies show that a number of species (including nonhuman apes, rhesus macaques, capuchin monkeys) seek information about the location of a hidden food reward before choosing one of the potential hiding places (sometimes interpreted as an indicator for metacognitive abilities; e.g., Call & Carpenter, 2001). The key finding here is that individuals predominantly seek information when they lack the relevant piece of information (i.e., where the food has been hidden). Recent studies showed that chimpanzees and orangutans also seek information about other objects such as tools (Bohn et al., 2017; Mulcahy, 2016). That is, when necessary to obtain a food reward, apes actively seek information about the location of the functional tool before making a choice. Whether they will also seek information to elucidate a knowledge-inconsistent rather than uncertain or unknown event remains unclear though. Put differently, in classic information seeking studies, the participants search for what / where information whereas explanation seeking paradigms aim at the question whether animals show an interest in *why* questions.
